# Top-down and bottom-up controls on southern New England salt marsh crab populations

**DOI:** 10.7717/peerj.4876

**Published:** 2018-05-30

**Authors:** Kenneth B. Raposa, Richard A. McKinney, Cathleen Wigand, Jeffrey W. Hollister, Cassie Lovall, Katelyn Szura, John A. Gurak, Jr., Jason McNamee, Christopher Raithel, Elizabeth B. Watson

**Affiliations:** 1 Narragansett Bay National Estuarine Research Reserve, Prudence Island, RI, United States of America; 2 ORD-NHEERL, Atlantic Ecology Division, U.S. Environmental Protection Agency, Narragansett, RI, United States of America; 3 Department of Biological Sciences, College of the Environment and Life Sciences, University of Rhode Island, Kingston, RI, United States of America; 4 The Scripps Research Institute, La Jolla, CA, United States of America; 5 Rhode Island Department of Environmental Management, Jamestown, RI, United States of America; 6 Rhode Island Department of Environmental Management, Kingston, RI, United States of America; 7 Department of Biodiversity, Earth and Environmental Sciences, Academy of Natural Sciences, Drexel University, Philadelphia, PA, United States of America

**Keywords:** New England, Community assessment, Sea-level rise, Predation, Crabs, Salt marsh

## Abstract

Southern New England salt marsh vegetation and habitats are changing rapidly in response to sea-level rise. At the same time, fiddler crab (*Uca* spp.) distributions have expanded and purple marsh crab (*Sesarma reticulatum*) grazing on creekbank vegetation has increased. Sea-level rise and reduced predation pressure drive these changing crab populations but most studies focus on one species; there is a need for community-level assessments of impacts from multiple crab species. There is also a need to identify additional factors that can affect crab populations. We sampled crabs and environmental parameters in four Rhode Island salt marshes in 2014 and compiled existing data to quantify trends in crab abundance and multiple factors that potentially affect crabs. Crab communities were dominated by fiddler and green crabs (*Carcinus maenas*); *S. reticulatum* was much less abundant. Burrow sizes suggest that *Uca* is responsible for most burrows. On the marsh platform, burrows and *Carcinus* abundance were negatively correlated with elevation, soil moisture, and soil percent organic matter and positively correlated with soil bulk density. *Uca* abundance was negatively correlated with *Spartina patens* cover and height and positively correlated with *Spartina alterniflora* cover and soil shear strength. Creekbank burrow density increased dramatically between 1998 and 2016. During the same time, fishing effort and the abundance of birds that prey on crabs decreased, and water levels increased. Unlike in other southern New England marshes where recreational overfishing is hypothesized to drive increasing marsh crab abundance, we propose that changes in crab abundance were likely unrelated to recreational finfish over-harvest; instead, they better track sea-level rise and changing abundances of alternate predators, such as birds. We predict that marsh crab abundance will continue to expand with ongoing sea-level rise, at least until inundation thresholds for crab survival are exceeded.

## Introduction

Southern New England salt marshes are undergoing rapid and dramatic change. Some conspicuous examples include the replacement of high marsh with low marsh vegetation, increasing areas of high marsh vegetation dieback, shallow pond formation, edge erosion, channel expansion, and soil weakening (Cole Ekberg, Ferguson & Raposa, 2015; Raposa et al., 2017a; Watson et al., 2017a). Sea-level rise is driving many of these changes, but other factors such as elevated nutrient levels, latent effects from hydrologic alterations, and plant fungal infections have also been identified as potential stressors (Deegan et al., 2012; Coverdale et al., 2013a; Elmer et al., 2013). Another emerging co-stressor, however, is impacts from crab populations (e.g., Holdredge, Bertness & Altieri, 2009).

Southern New England marshes have typically supported crab communities comprised of few species at relatively low densities. One early survey in a Rhode Island (RI) salt marsh documented a crab community dominated by the mud fiddler crab, *Uca pugnax*, followed by the purple marsh crab, *Sesarma reticulatum* (hereafter *Uca* and *Sesarma*, respectively), which was common but not abundant, and two additional *Uca* species that were very rare (Bertness & Miller, 1984). In later studies, the European green crab, *Carcinus meanas* (hereafter *Carcinus*) emerged as a common component of crab communities in both subtidal and marsh surface habitats (Roman et al., 2002; Meng, Cicchetti & Chintala, 2004). Early research also demonstrated that *Uca* burrowing enhances *Spartina alterniflora* production by aerating soils and promoting drainage (Bertness, 1985) and, to our knowledge, reports of negative impacts to marshes from crabs were absent in southern New England up through the early 2000s. However, observations of expanding *Uca* and *Sesarma* populations have proliferated across the region within the last decade, and studies are now confirming negative impacts to marshes across the region associated with excessive crab burrowing and grazing (Holdredge, Bertness & Altieri, 2009; Coverdale, Bertness & Altieri, 2013b; Schultz, Anisfeld & Hill, 2016).

Many reports linking marsh degradation to crabs have emerged from Cape Cod MA, where Holdredge, Bertness & Altieri (2009) first demonstrated a link between the loss of low marsh *S. alterniflora* and excessive *Sesarma* herbivory, which was in turn linked to a reduction in predation pressure. A series of subsequent studies further demonstrated that *Sesarma* overgrazing (1) ultimately stems from recreational overfishing, (2) negatively impacts multiple ecosystem services, (3) creates denuded areas on the marsh surface favored by *Uca* and that facilitate *Uca* expansion, and (4) can be mitigated by the invasive crab *Carcinus* (Altieri et al., 2012; Bertness & Coverdale, 2013; Brisson, Coverdale & Bertness, 2014; Coverdale et al., 2014; Smith, 2015). In Connecticut, Luk & Zajac (2013) documented an intra-marsh range expansion of *U. pugnax* and attributed it to sea-level rise. These findings expand the geographic extent of impacts to marshes from crabs. For example, the headward erosion of creek networks in Georgia and South Carolina has been linked to excessive crab burrowing in association with sea-level rise (Hughes et al., 2009; Wilson, Hughes & FitzGerald, 2012; Vu, Wieski & Pennings, 2017), and in southern Maine the physical removal of low marsh *S. alterniflora* is occurring even in the absence of *Sesarma*, and is instead being linked to recent increases in *Carcinus*, which are burrowing into vertical creekbanks (Belknap & Wilson, 2014).

In RI, new research is now documenting vegetation shifts, seaward edge erosion, drainage channel expansion, and the net loss of marsh area (Watson et al., 2017a), but few studies have linked these changes to crabs despite proliferating anecdotal reports of localized high *Uca* densities in marshes. As late as 2007, Holdredge, Bertness & Altieri (2009) reported that creekbank vegetation loss and *Sesarma* densities were very low in RI compared to Cape Cod marshes. By 2013, however, Bertness et al. (2014) documented an increase in creekbank vegetation loss over time in some RI marshes and attributed this to increased *Sesarma* gazing due to overfishing as in Cape Cod. In fact, Coverdale, Bertness & Altieri (2013b) report that this is a region-wide phenomenon that is occurring at least from Long Island Sound to Massachusetts. The evidence linking creekbank vegetation loss to *Sesarma* grazing in RI is compelling, but the sea-level rise-induced *Uca* expansion in CT, reports of creekbank vegetation loss in Maine linked to *Carcinus*, and reports of high *Uca* densities across RI call for investigations into the potential role of other crab species in addition to *Sesarma*. Moreover, it is difficult to definitively link marsh changes over time in RI to increasing crab populations, or to specific crab species, because time-series data on crab abundance is generally lacking and because no recent studies have considered all marsh crab species simultaneously.

The above-mentioned studies demonstrate that crab populations are impacting salt marshes across New England and identify sea-level rise and overfishing of crab predators as drivers of expanding crab populations in some locations. However, more research is needed to quantify the relative degree of impacts to marshes from multiple crab species and to identify environmental parameters that affect the distribution and abundance of each species. We therefore conducted a field study in 2014 to examine relationships between crab populations and vegetation, elevation, and soil characteristics among marshes and habitat types. We also included a second study component to quantify recent temporal trends in crab abundance and a suite of potential drivers of crabs. The specific goals of this study were to (1) describe current marsh crab community and population demographics, (2) quantify patterns in crab species among marshes and habitats, (3) identify significant correlates of crab abundances in multiple marsh habitats, and (4) quantify temporal trends in crab abundance and potential drivers of change. Our study provides insight into the current composition, distribution, and habitat use of marsh crabs in RI and serves as a baseline to which future studies can be compared to assess change. It provides additional insight into the potential causes and effects of changes in crab abundance over time in southern New England salt marshes. Most studies focus either on *Uca* or *Sesarma* individually; here we focus on all dominant crab species simultaneously to provide a holistic assessment of crab dynamics in RI marshes.

## Materials & Methods

### Study sites

**Figure 1:**
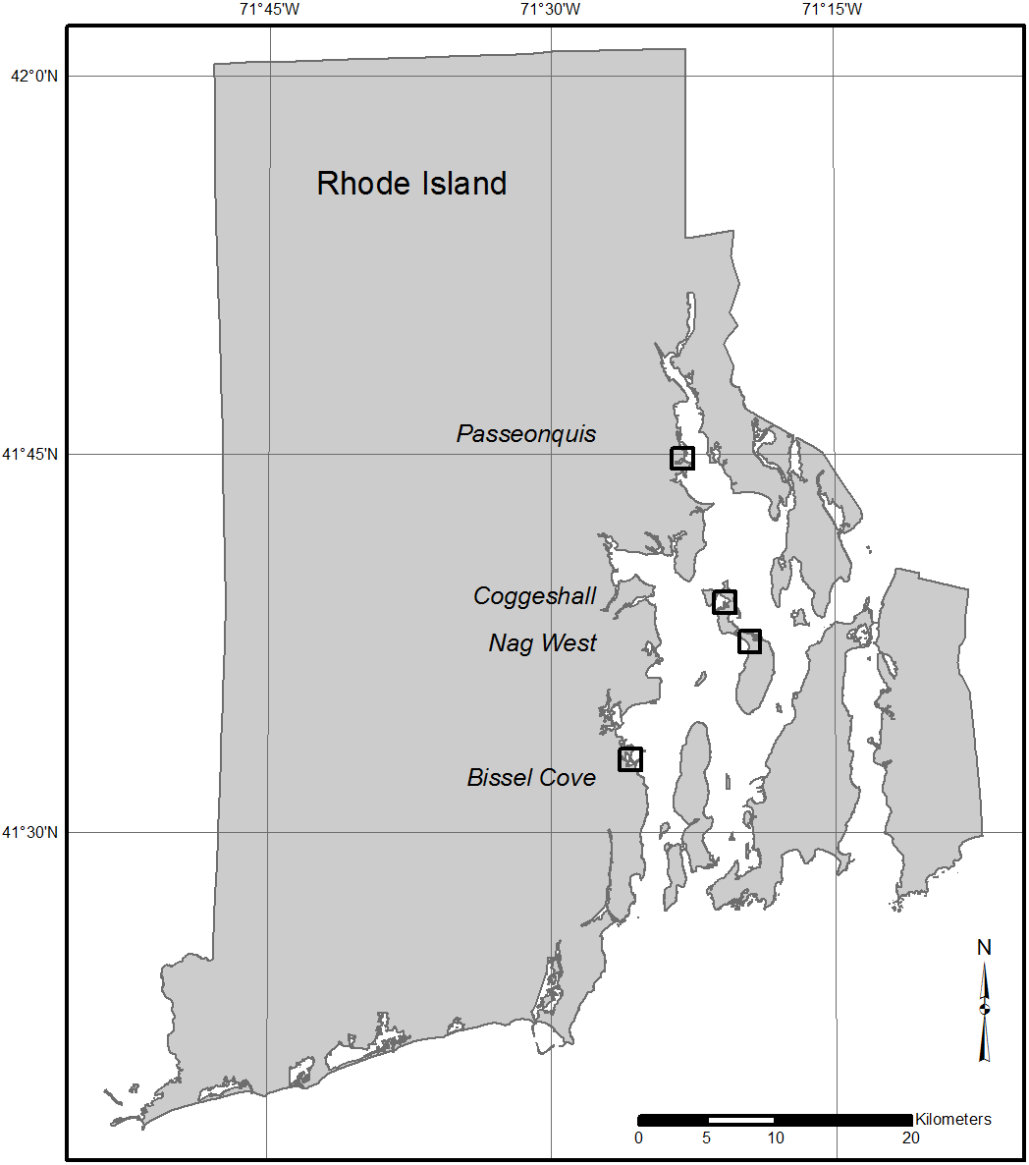
Locations of the four study marshes in Narragansett Bay, RI. The marshes include Bissel Cove Marsh (BIS), Coggeshall Marsh (COG), Nag Marsh (NAG), and Passeonquis Marsh (PAS).

This study was conducted at four salt marshes selected to represent a broad range of conditions along known elevation and habitat composition gradients within Narragansett Bay, RI (Cole Ekberg, Ferguson & Raposa, 2015). The sites included Bissel Cove Marsh (BIS; North Kingstown), Coggeshall Marsh (COG; Prudence Island and in the Narragansett Bay National Estuarine Research Reserve [NBNERR]), Nag Marsh (NAG; Prudence Island, NBNERR), and Passeonquis Marsh (PAS; Warwick) (Table 1; Fig. 1). Vegetated habitats were typical of southern New England salt marshes (e.g., Warren & Niering, 1993), but the relative amount of dominant species in each marsh followed a predictable pattern in relation to overall mean site elevation: as site elevation increased, the percent composition of flood-tolerant *S. alterniflora* decreased, and the composition of less flood-tolerant high marsh species (e.g., *S. patens*, *Distichlis spicata*) increased. All marshes, however, exhibited impacts that have been linked to sea-level rise and/or crabs, including the presence of bare creekbanks and the net loss of marsh area over time (Bertness et al., 2014; Watson et al., 2017a).

**Table 1:** Characteristics of the four study sites. Marsh type from McKinney & Wigand (2006); coordinates from Watson et al. (2017a); area from Raposa et al. (2017a; COG, NAG) and Watson et al. (2017a; BIS, PAS); tide range from Raposa et al. (2016; COG, NAG) and Wigand et al. (2003; BIS, PAS); elevation from Watson et al. (2017a).

	BIS	COG	NAG	PAS
Type	Wide fringe	Meadow/fringe	Salt-meadow	Wide fringe
Latitude	41°16′N	41°39′N	41°37′N	41°46′N
Longitude	71°26′W	71°20′W	71°19′W	71°24′W
Area (ha)	3.3	25.5	15.3	3.6
Tide range (m)	0.87	1.16	0.53	0.8
Median elevation (m NAVD88)	0.36	0.62	0.64	0.75

**Notes.**

BIS Bissel Cove Marsh COG Coggeshall Marsh NAG Nag Marsh PAS Passeonquis Marsh

### Field sampling

We conducted stratified random sampling, establishing five random sampling stations in each of four habitat types, for a total of 20 stations per marsh. Once randomly selected, station locations remained fixed and were permanently marked with a PVC stake. Habitats included (1) bare/unvegetated creekbanks (BCB), (2) vegetated creekbanks (VCB), (3) the marsh platform (MP), and (4) the *Iva frutescens* marsh/upland transition zone (IVA). All bare and vegetated creekbank stations were located within ∼1 m of a creek edge, and all vegetated creekbank stations were in tall-form *S. alterniflora*. The marsh platform included the entire high marsh plain between the low marsh and *I. frutescens* zones (i.e., including short *S. alterniflora*, *S. patens*/*D. spicata* salt meadow, *Juncus gerardii*/*D. spicata* high marsh, and dieback pannes). No stations were located in open water features such as creeks, pools, or ditches.

Marsh crabs were sampled at all stations three times in summer 2014 (approximately monthly, June through August) using two different methods that are commonly used to sample marsh crabs or provide surrogate estimates of crab abundance. We used a 0.5 m ×0.5 m quadrat sampler to conduct crab burrow counts. Around low tide, the quadrat was placed in the same location relative to each plot’s locator stake. All crab burrows were then counted and their diameters measured. We counted all burrows that were >3 mm in diameter in order to include all crab species. We also sampled crabs with pitfall traps. The traps were 7.5 cm in diameter by 20 cm deep with a capped bottom and perforated sides for drainage. Each trap was pushed into the marsh peat until the top of the trap was flush with the marsh surface. To deploy, the cap of each trap was removed around low tide and the trap was allowed to fish for 24 h. Upon retrieval, all captured crabs were removed from each trap, identified to species, measured (carapace width), and released. From burrow counts we calculated crab burrow density (number m^−2^); from pitfall traps we calculated catch-per-unit-effort (CPUE; an indicator of abundance, with effort being the number of traps) for each species.

Ancillary environmental parameters were also sampled once at each station in summer 2014. We collected data on elevation, vegetation communities, and edaphic conditions (soil bulk density, percent soil moisture, percent soil organic matter, and soil shear strength). Plot elevation was measured relative to tidal and orthometric datums. At COG and NAG, plots were surveyed relative to National Geodetic Survey benchmarks whose elevations (relative to NAVD88) were established using GPS static post-processed kinematic surveys (PPK). At BIS and PAS, several temporary benchmarks (PVC pipes topped with survey markers driven to refusal) were established per marsh, and elevations of these benchmarks were measured using PPK GPS surveys. Differential leveling was conducted using a Self-Leveling Exterior Rotary Laser (CST/Berger, Watseka, IL, USA), with an accuracy of ±1.5 mm at 30 m. The elevation of each plot was estimated as the mean of five points: the four corners and center of each quadrat. Plot elevations were also calculated relative to mean high water (MHW) for the 1983–2001 National Tidal Datum Epoch using the VDatum vertical transformation tool. VDatum converts between orthometric, tidal, and ellipsoidal datums, and interpolates tidal datums between tide gauges using hydrodynamic model simulations (ADCIRC) run using nearshore bathymetric data (Yang et al., 2008). While the maximum cumulative uncertainty reported for converting between NAVD88 and MHW for the RI region is 3.0 cm, VDatum documentation cautions that larger errors were typically seen in marshes and in areas where the tides change rapidly, such as in barrier estuaries and upriver sites (NOAA, 2015).

Within the burrow count quadrats, we quantified the percent cover of all plant species (and other relevant cover types such as bare ground) using the point-intercept technique at 50 grid points (Roman, James-Pirri & Heltshe, 2001). When any dominant marsh grass (*S. alterniflora*, *S. patens*, *D. spicata*, *J. gerardii*) was present in a quadrat, the heights of up to 12 random plants of each species (three from each corner of the quadrat) were also measured. We used a geovane to twice measure marsh soil strength every 10 cm to 1 m depth or to refusal in each quadrat. We collected a surface soil sample (usually 5 cm × 5 cm) between plant shoots to a depth of 4 cm with a sharp knife in each quadrat. The soil sample was collected ±2 h of low tide. Soil moisture (wet wt. [g] − dry wt. [g]), bulk density (dry wt. [g]/volume [mL]), and percent organic matter were determined for each sample. Samples were dried at 50°C until a constant weight was attained (usually 48 h). Dried samples were ashed at 550°C for 6 h to determine percent organic matter (Heiri, Lotter & Lemcke, 2001).

### Temporal trends

Existing monitoring and survey data were compiled and augmented with new data collected in this study to quantify temporal trends in crab abundance (i.e., burrow density), creekbank vegetation loss, and a variety of factors that can potentially affect crab abundance. Water level change was the bottom-up factor; top-down factors included recreational fishing pressure and abundances of two levels of higher-order predators (wading birds as direct crab predators; ospreys as apex predators of fish that prey on crabs).

Burrow density (number m^−2^) in creekbank and marsh platform habitats was compared between 1998 and 2016 using counts from three randomly-placed 0.25 m^−2^ quadrats in each habitat each year in ten reference salt marshes (Wigand et al., 2010) across Narragansett Bay. Within each quadrat, all burrow holes were counted; we did not use destructive excavations to determine which species occupied each burrow or which burrows were currently active. Trends in the extent of bare creekbanks were calculated from ongoing habitat monitoring along multiple transects each in COG and NAG (Raposa & Weber, 2013). Recreational fishing statistics were obtained for 2004–2014 from the Marine Recreational Information Program (MRIP) online data query tool (http://www.st.nmfs.noaa.gov/st1/recreational/queries/). The species chosen for analysis were striped bass (*Morone saxatilis*), scup (*Stenotomus chrysops*), black sea bass (*Centropristis striata*), and tautog (*Tautoga onitis*) as all of these species are known crustacean predators (Bigelow & Schroeder, 1953) and are important recreational species in RI (Shepherd & Nieland, 2010; ASMFC, 2015a; ASMFC, 2015b; Northeast Fisheries Science Center , NEFSC). Trends in the amount of fishing effort over time were calculated for all species combined and for the marsh-associated striped bass only. Effort was defined as directed angler trips taken with the species above as the primary or secondary target. Trends in the number of wading bird nests in coastal RI (an indicator of wading bird abundance) were calculated from the RI Department of Environmental Management state-wide monitoring program. Trends were calculated for all wading bird species that forage in RI salt marshes combined (Black-crowned Night Heron (*Nycticorax nycticorax*), Cattle Egret (*Bubulcus ibis*), Glossy Ibis (*Plegadis falcinellus*), Great Egret (*Ardea alba*), Little Blue Heron (*Egretta caerulea*), and Snowy Egret (*Egretta thula*)) and on a subset that commonly prey on crustaceans (Glossy Ibis and Black-crowned Night Heron). Trends in the number of osprey (*Pandion haliaetus*) nests in coastal RI (an indicator of osprey abundance) were calculated from the Audubon Society of Rhode Island’s ongoing annual monitoring program. Finally, trends in annual mean high water (MHW; referenced to the North American Vertical Datum of 1988) were calculated from monthly MHW data obtained from the Newport RI NOAA tide station (ID# 8452660; https://tidesandcurrents.noaa.gov/).

The duration of each of these temporal datasets varied somewhat due in part to data availability, but we truncated most to span from 1998 (the first burrow density survey) to 2014 (when the bulk of the field data were collected for this study). Exceptions include (1) burrow density, which was re-sampled in 2016, (2) bare creekbank extent, which was extended to 2015 to help reveal trends since this habitat was not detected until 2012 in our sites, and (3) recreational fishing data, which were not readily available before 2004 due to a statistical survey design change.

### Data analysis

We used burrow density, and *Uca*, *Carcinus*, and *Sesarma* CPUE as indicators of crab abundance in this study, and derived crab and burrow size distributions to further describe crab demographics. Patterns in crab indicators were examined among marshes, among habitats, and in association with environmental parameters within each habitat. For each indicator, two-way ANOVAs were run to detect differences among the four marshes and four habitats. ANOVA models were run on rank-transformed data because raw and log-transformed data did not meet the assumptions for a strictly parametric test (multiple comparisons require significant subsetting of the data and, for this analysis, results in very small sample sizes per comparison; multiple comparisons were therefore not run). Pearson correlation tests for bivariate pairs with 10 or more available samples were used to evaluate relationships between each crab indicator and environmental parameters within each of the four habitats using data pooled among the four marshes. Both significant and non-significant correlations are reported. Paired *t*-tests were used to compare burrow density between 1998 and 2016 in creekbank and marsh platform habitats. Temporal trends for all remaining factors were quantified using simple linear regression on annual data over time. All statistical analyses were performed with R version 3.4.0 (Chang, 2009; Wickham, 2016; Hollister & Raposa, 2017; R Core Team, 2017; Revelle, 2017; Robinson, 2017; Rudis, 2017; Wickham, 2017; Wickham & Bryan, 2017; Wickham & Henry, 2017; Wickham et al., 2017a; Wickham, Hester & Francois, 2017b). Code and data for all analyses are available via GitHub (https://github.com/jhollist/crabs) and archived on Zenodo (Hollister & Raposa, 2017).

## Results

### Crab community and population characteristics

**Figure 2:**
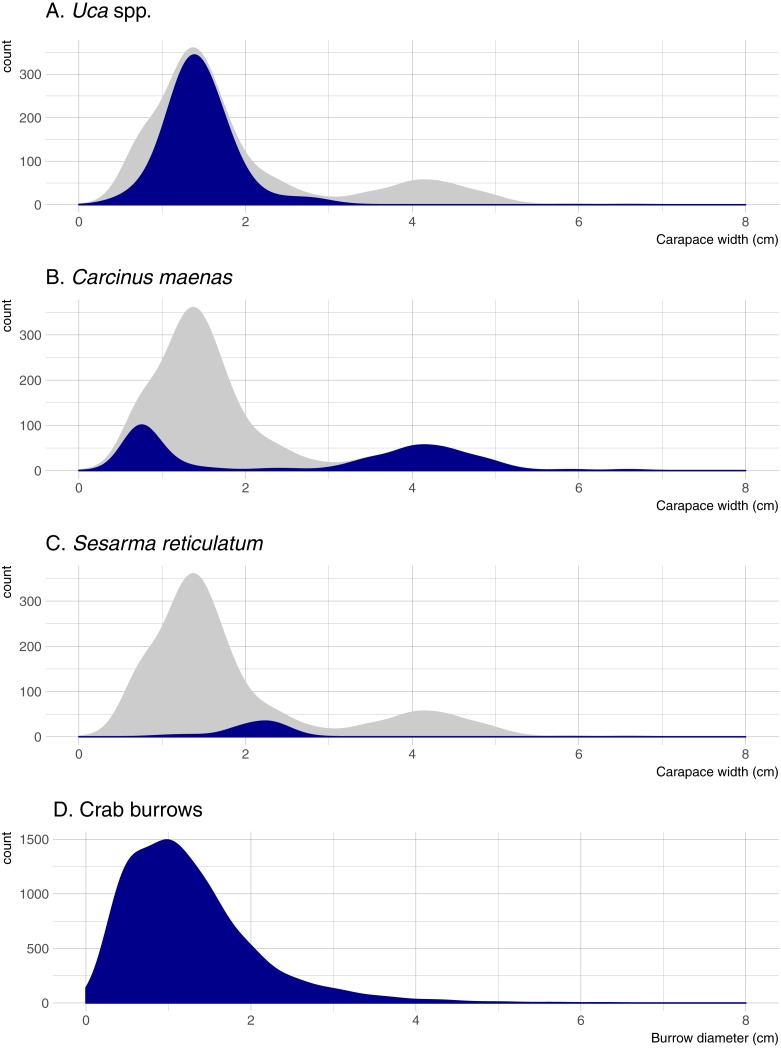
Density distributions of crab burrow diameters and carapace widths for *Uca*, *Carcinus*, and *Sesarma*. Grey distribution represents all crab species. Note that *y* axes are at different scales. (A) *Uca* spp. (B) *Carcinus maenas*. (C) *Sesarma reticulatum*. (D) Crab burrows.

Five crab species were captured in pitfall traps during this study. *Uca pugnax* was by far the most abundant species (318 captured; 64.5% of all crabs), followed by *C. maenas* (146; 29.6%), *S. reticulatum* (27; 5.5%), one sand fiddler crab *Uca pugilator* (0.2%), and one unidentified mud crab (family Xanthidae; 0.2%). Hereafter, the two fiddler crab species will be pooled and referred to as *Uca*. Nearly all captured *Uca* were less than 2-cm wide (carapace width) and averaged 1.45 cm overall; *Sesarma* were slightly larger and averaged 2.10 cm (Fig. 2). *Carcinus* exhibited a bimodal size distribution with a sharp peak in juveniles that were less than 1-cm wide (mostly from one pitfall sample) and another peak of adults ranging from 3.5 to 5.0-cm wide. Mean crab burrow densities ranged from 0 to 257 m^−2^ at individual plots (overall mean = 42 burrows m^−2^), and burrow diameters ranged from 0.3 to 9.0 cm (mean = 1.3 cm). Most burrows (87%) were less than 2 cm in diameter (Fig. 2), which is typically the upper size threshold for *Uca* (Bertness, Holdredge & Altieri, 2009); only 7.9% of burrows were ≥ 2.5 cm diameter, the reported minimum threshold for *Sesarma* (Bertness, Holdredge & Altieri, 2009).

### Variability among marshes and habitats

**Figure 3:**
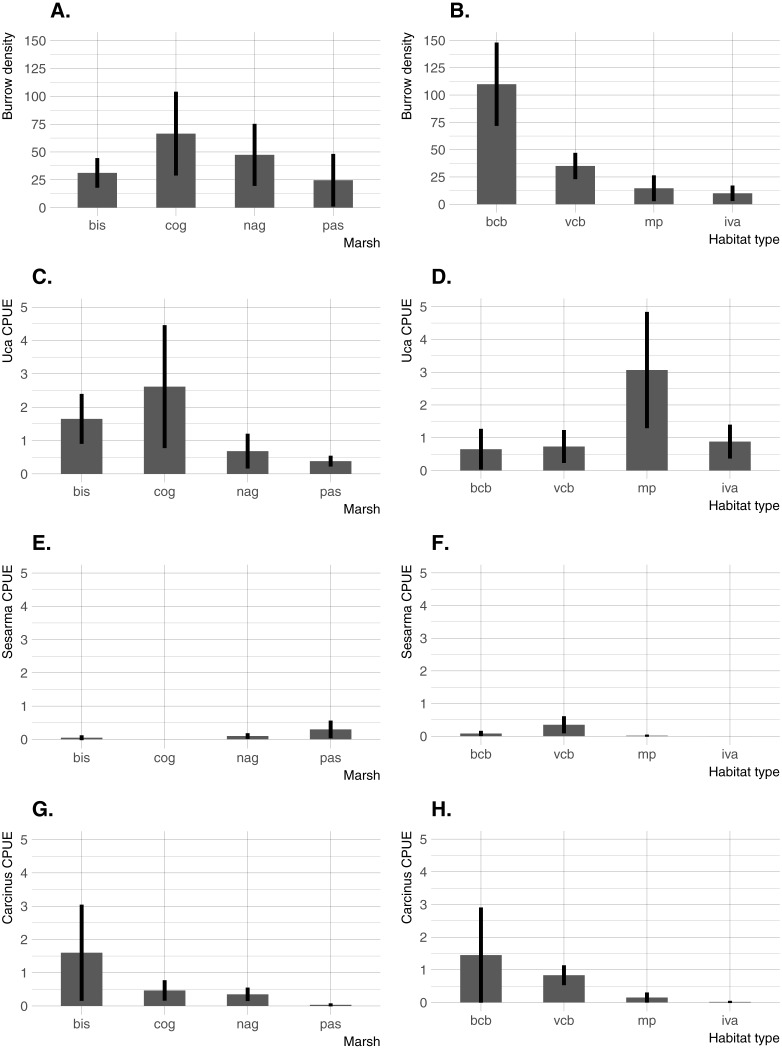
Burrow density and crab species CPUE across the four study sites and four habitats. (A) Burrow density among marshes. (B) Burrow density among habitats. (C) *Uca* CPUE among marshes. (D) *Uca* CPUE among habitats. (E) *Sesarma* CPUE among marshes. (F) *Sesarma* CPUE among habitats. (G) *Carcinus* CPUE among marshes. (H) *Carcinus* CPUE among habitats. Error bars are 95% confidence limits and thus may be compared with each other to imply significance. BIS, Bissel Cove Marsh; COG, Coggeshall Marsh; NAG, Nag Marsh; PAS, Passeonquis Marsh; BCB, bare creekbanks; VCB, vegetated creekbanks; MP, marsh platform; IVA, Iva frutescens zone.

Significant differences in all crab indicators were found among marshes, among habitats, and for marsh/habitat interactions (main ANOVA results are summarized in Table 2). Among marshes, burrow density was highest at the two intermediate-elevation sites (COG and NAG; median elevation =0.62 − 0.64 m NAVD88), whereas *Uca* CPUE was highest at the lowest-elevation site (BIS; median elevation = 0.36 m NAVD88) and one intermediate-elevation site (COG) (Fig. 3). *Carcinus* CPUE and *Sesarma* CPUE were highest at the lowest (BIS) and highest (PAS) elevation marsh, respectively. Among habitats, burrow density was by far highest in bare creekbanks, and *Uca* CPUE on the marsh platform. *Carcinus* and *Sesarma* CPUE were both highest in the two types of creekbank habitats.

**Table 2:** Summary of main ANOVA results comparing indicators of crab abundance among four study marshes and among four different habitat types within each marsh.

Indicator	Source	*df*	*F*	*p*
Burrow density	Marsh	3	4.20	0.009
	Habitat	3	21.03	<0.001
	Marsh × habitat interaction	9	2.72	0.010
*Uca* CPUE	Marsh	3	5.10	0.003
	Habitat	3	6.72	0.001
	Marsh × habitat interaction	9	3.64	0.001
*Carcinus* CPUE	Marsh	3	14.58	<0.001
	Habitat	3	24.62	<0.001
	Marsh × habitat interaction	9	2.21	0.033
*Sesarma* CPUE	Marsh	3	5.10	0.003
	Habitat	3	8.91	<0.001
	Marsh × habitat interaction	9	3.79	0.001

### Crab/environmental correlations within habitats

**Figure 4:**
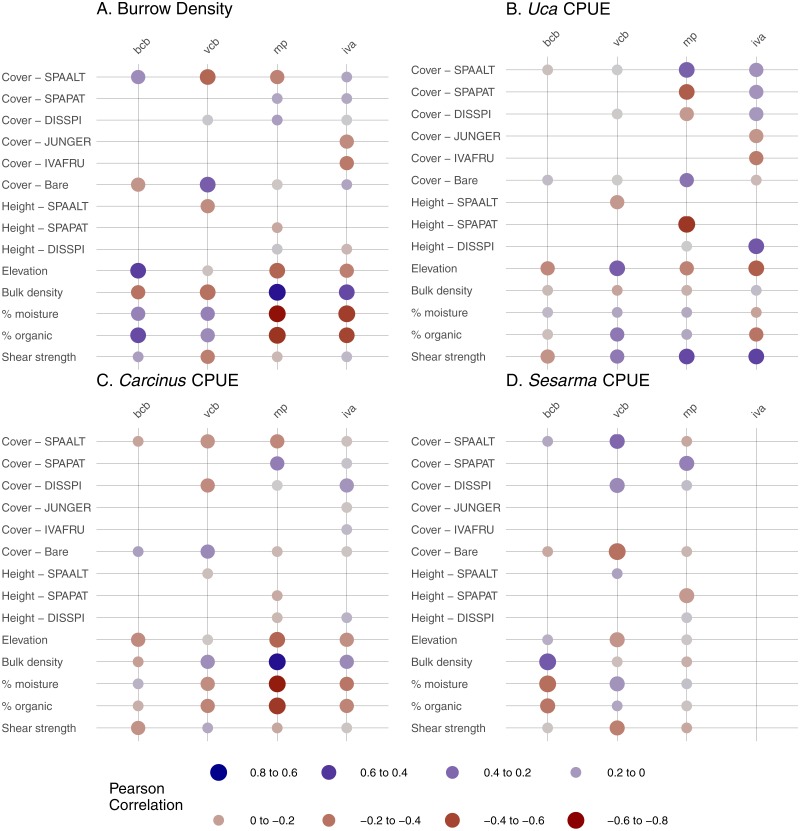
Correlations between crab abundance indicators and environmental parameters in four habitat types. Correlations were run for pairs with 10 or more samples. BCB, bare creekbanks; VCB, vegetated creekbanks; MP, marsh platform; IVA, *Iva frutescens* zone; SPAALT, *Spartina alterniflora*; SPAPAT, *Spartina patens*; DISSPI, *Distichlis spicata*; JUNGER, *Juncus gerardii*; IVAFRU, *Iva frutescens*. (A) Burrow Density (B) *Uca* CPUE (C) *Carcinus* CPUE (D) *Sesarma* CPUE.

Crab burrow density correlated with multiple edaphic indicators on the marsh platform and in the *Iva* zone (Fig. 4). In general, more burrows were found where soil bulk density was high, and where percent organic matter and percent moisture were low. In vegetated creekbanks, burrow density correlated negatively with *S. alterniflora* cover and positively with bare cover. Burrows also strongly correlated with elevation—positively in bare creekbanks and negatively on the marsh platform.

*Uca* CPUE correlated strongly with *S. patens* cover and height (negative correlation) and *S. alterniflora* cover (positive) on the marsh platform (Fig. 4), demonstrating a negative association with dense, tall *S. patens* in favor of short-form *S. alterniflora*. *Uca* CPUE was also positively correlated with soil shear strength on the marsh platform and in the *Iva* zone. *Carcinus* CPUE was positively related to soil bulk density and negatively related to elevation, soil percent moisture, and soil percent organic matter on the marsh platform (Fig. 4), showing that *Carcinus* is most common in low-elevation areas with relatively dry, compact, mineral soils when on the marsh platform. The strongest relationship for *Sesarma* CPUE was a positive correlation with soil bulk density in bare creekbanks.

**Figure 5:**
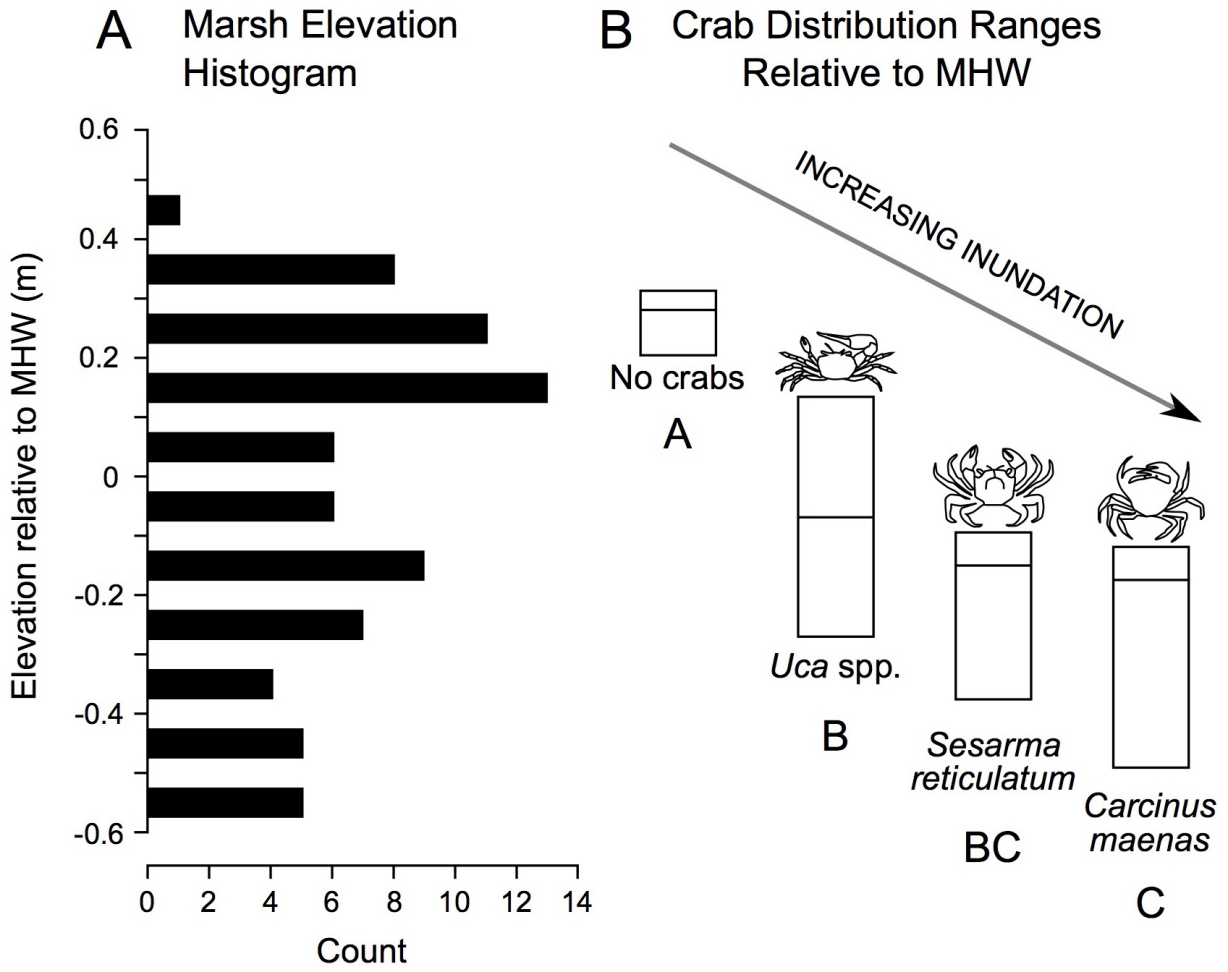
Distribution of study plot elevations relative to mean high water (A), and elevation distributions (inter-quartile ranges) of crab species caught in pitfall traps (B). No crabs were captured at the highest marsh elevations. At mid-marsh elevations, *Uca* was found; *Sesarma* and *Carcinus* were caught at lower elevations. Different subscripts among species indicate significant differences in mean elevation based on ANOVA and a Games Howell pairwise test (*F* = 10.225, *p* < 0.001). These data suggest that as coastal marsh inundation increases with sea-level rise, shifts from no crabs to *Uca* and then to *Sesarma* and *Carcinus* are expected.

In addition to correlations, we derived elevation range distributions for each crab species. These data reveal a gradient in crab distribution across elevation, with no crabs found at highest elevations, followed in order by *Uca*, *Sesarma*, and *Carcinus* as elevation declines (Fig. 5).

### Temporal trends

**Figure 6:**
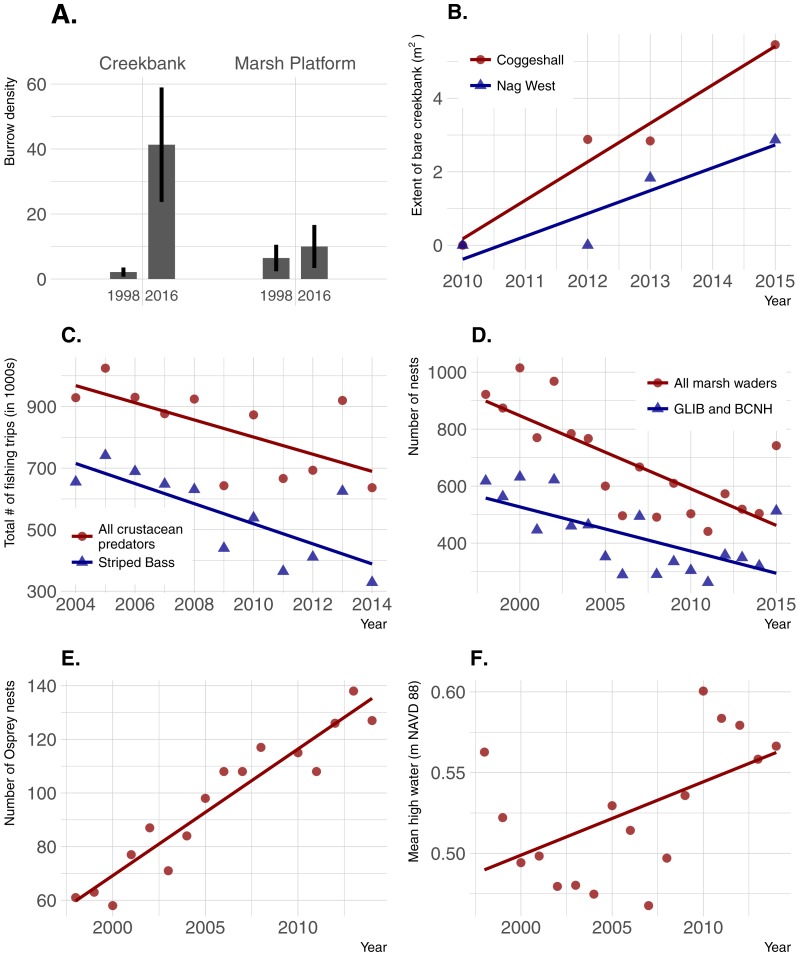
Temporal changes in crab abundance (burrow density), a habitat change linked to crabs (extent of bare creekbanks), and factors that can potentially affect crab abundance. (A) Burrow density; error bars are 95% confidence limits. (B) Extent of bare creekbanks. (C) The level of recreational fishing, defined as directed trips with primary and secondary targets of crustacean predators. (D and E) Predation pressure by birds. (F) Sea-level rise.

Burrow density increased dramatically over time in creekbanks (*t* =  − 4.44; *p* = 0.0001) but not on the marsh platform (*t* =  − 0.909; *p* = 0.367) (Fig. 6). Bare creekbanks were absent from both NBNERR marshes in 2010, but have been increasing linearly in COG since appearing in 2012 (*R*^2^ = 0.96; *p* = 0.02), and trending higher since appearing in NAG in 2013 (*R*^2^ = 0.83; *p* = 0.09). Recreational fishing effort decreased between 2004 and 2014, and this decrease was consistent for all major crustacean-eating sport fish (*R*^2^ = 0.43; *p* = 0.03), and for the marsh-associated striped bass only (*R*^2^ = 0.58; *p* < 0.01). The declines in fishing pressure coincide with steady to increasing stock numbers for all of these same fish species in Narragansett Bay (S Olszewski, 2017, unpublished data; Project No. F-61-R-21, available upon request from RIDEM Division of Fish and Wildlife Marine Fisheries). Conversely, wading bird abundance has declined in RI since 1998 (*R*^2^ = 0.57; *p* < 0.001 for all wading bird species; *R*^2^ = 0.44; *p* = 0.003 for Glossy Ibis and Black-crowned Night Heron only), and osprey abundance has increased significantly since 1998 (*R*^2^ = 0.90; *p* < 0.0001). Finally, annual MHW increased significantly from 1998–2014 (*R*^2^ = 0.29; *p* = 0.03).

## Discussion

Our results from four marshes show that *Uca* dominates crab communities in RI marshes and that most creekbank burrows (87%) are sized for *Uca*. The significant increase in burrow density over the past ∼15 years is not coincident with an increase in recreational fishing pressure as reported elsewhere (e.g., Altieri et al., 2012); instead it more closely tracks rising sea levels and changing populations of alternate predators. Our data also demonstrate strong correlations of crabs with vegetation, elevation, and soil characteristics; because these same environmental factors are known to change with sea-level rise (e.g., shifts from high to low marsh vegetation, loss of elevation capital, wetter soils; Cole Ekberg, Ferguson & Raposa, 2015; Raposa et al., 2017a; Watson et al., 2017b), our data and results can therefore be used to help predict how crab communities will respond as sea-level rise accelerates*.*

### Crab patterns among marshes and habitats

All four crab indicators (burrow density, and *Uca*, *Carcinus*, and *Sesarma* CPUE) varied significantly among marshes, further demonstrating that crabs and their potential impacts can vary considerably from marsh to marsh even in a relatively small geographic area (Holdredge, Bertness & Altieri, 2009; Bertness et al., 2014). The distribution and relative abundance of crabs also varied significantly among habitats in our study. *Sesarma* and *Carcinus* were concentrated in low-elevation creekbank habitats, but *Uca* was found in the entire range of marsh habitats from bare creekbanks up through the *Iva* zone. This is consistent with the findings of Luk & Zajac (2013), who documented a within-marsh *Uca* range expansion and attributed it as a response to sea-level rise, and with Vu, Wieski & Pennings (2017), who found *Sesarma* concentrated within creek heads and *Uca* common throughout the marsh. In our study, *Uca* CPUE was actually highest on the marsh platform. This was somewhat surprising given that burrow density (most of which were *Uca* burrows) was highest along creekbanks, and that *Uca* was consistently observed in very high densities on bare creekbanks throughout the study (K Raposa & R McKinney, pers. obs., 2014). Multiple factors may have contributed to this disconnect, including a possible overestimation of active burrows in creekbanks and the fact that *Uca* frequently forages considerable distances from their burrows and can exhibit poor burrow fidelity (Montague, 1980; Bertness & Miller, 1984). Another contributing factor may be our choice of pitfall traps as sampling gear. Although pitfall traps are commonly used to sample salt marsh crabs (e.g., Altieri et al., 2012), there are known issues associated with this sampling gear, both in terrestrial and aquatic habitats (Topping & Sunderland, 1992; Fanini & Lowry, 2016). Pitfall traps have not been fully evaluated in salt marshes, but we suspect that data can be affected by factors such as the presence of predators in traps (i.e., *Carcinus* in creekbanks) and the lack of trap drainage in waterlogged soils that may facilitate crab escape. In general, care should be taken when interpreting results from any study that relies on data collected only from pitfall traps, at least until the effectiveness of this sampling gear is further evaluated in salt marshes. Regardless, our results unambiguously demonstrate that *Uca* currently utilizes a wide range of marsh habitats that includes high elevation areas in the *Iva* zone. This aligns well with Teal (1958), who also found *U. pugnax* distributed throughout a Georgia salt marsh, suggesting that some faunal community patterns in southern New England marshes may now be trending towards similarity with southeastern US marshes.

Similar to *Uca*, crab burrows were also found in all habitats in our study. Burrow density was higher in bare creekbanks compared to all other habitats, and trended lower while moving away from creekbanks towards the upland/marsh edge. In bare creekbanks, burrow density averaged 110 m^−2^ and peaked above 250 m^−2^. These very high densities result in “Swiss-cheese” creekbanks that are similar in appearance to other marshes in the region that are heavily impacted by *Sesarma* (Schultz, Anisfeld & Hill, 2016). With our data it is not possible to determine whether dense creekbank crab burrows preceded creekbank vegetation loss or vice versa, but other studies demonstrate a negative impact on marsh vegetation from dense crab burrowing (Wilson, Hughes & FitzGerald, 2012). Even if crab burrowing is not the direct cause of creekbank vegetation loss in RI marshes, dense burrow networks are surely accelerating the rate of bank undercutting and erosion.

### Correlations with environmental parameters

The distribution and abundance of crabs in RI salt marshes was affected by multiple environmental parameters whose relative influence varied by species and habitat. By far most of the strongest correlations were between crab indicators and edaphic conditions (12 of the 14 correlations greater than an absolute value of 0.5). This is consistent with other studies that demonstrate a strong effect of soil condition and strength on crabs and burrowing (e.g., Bertness & Miller, 1984; Jaramillo & Lunecke, 1988). In general, crabs tend to burrow in soils of intermediate strength; soils that are too weak are less able to support burrow walls, and those that are too firm inhibit burrowing (Bertness & Miller, 1984). However, in our study all four crab indicators were positively correlated with high soil shear strength and/or high bulk density in at least one habitat type. We interpret these findings as a sign that soils in our study marshes have generally weakened (e.g., from habitat shifts and waterlogging; Wigand et al., 2014; Cole Ekberg, Ferguson & Raposa, 2015) to the point where crabs and burrows are now concentrated in areas where soil strength remains relatively high. Additionally, the common occurrence of crab burrows in sandier soils in our marshes may be attributed to drowning soils at low elevations being too waterlogged for successful burrow construction.

There were surprisingly few strong correlations between crabs and vegetation within each habitat zone. Notable exceptions were the increase in burrow density with decreasing vegetation cover along creekbanks, and decreasing *Uca* CPUE with increasing *S. patens* cover and height on the marsh platform. The former is consistent with the higher burrow densities in bare creekbanks compared to vegetated creekbanks described above. The latter supports previous studies that demonstrate inhibited *Uca* activity and abundance in the high marsh due to thick, healthy *S. patens* (Bertness & Miller, 1984). Given that *S. patens* is in rapid decline in many RI marshes due to sea-level rise (Watson et al., 2016; Raposa et al., 2017a), we hypothesize that *Uca* populations will benefit if rates of RI marsh elevation gain continue to lag behind sea-level rise (Raposa et al., 2017b). Elevation was also a minor correlate of crab burrow density and CPUE, at least at the within-habitat scale, but two relationships suggest upper and lower elevation bounds for crabs in marshes. The positive correlation between burrow density and elevation in bare creekbanks suggests that creekbanks lose their capacity to support burrows and crabs with declining elevation. Likewise, the negative correlation between *Uca* CPUE and elevation in the *Iva* zone suggests that there is an ultimate high-elevation threshold for this species in marshes.

Ultimately, the relationships between crabs, habitats, and environmental parameters will help inform how crab populations will respond to future sea-level rise. We predict that *Sesarma* and *Carcinus* will expand into previously inaccessible marsh interiors based on their association with creekbank habitats that are expanding in response to sea-level rise (Watson et al., 2017a). Our correlation analyses suggest contrasting responses by crabs to varying environmental changes associated with sea-level rise. For example, the shift from *S. patens* to *S. alterniflora* (Watson et al., 2016; Raposa et al., 2017a) should favor *Uca* and crab burrowing on the marsh platform, and pioneering *Uca* should further expand landward as its upper elevation bound increases with sea-level rise. In contrast, overall crab burrowing may eventually become inhibited if soils become too weak or waterlogged as marsh inundation progresses. When combined with the overall species-specific elevation ranges documented in our study (Fig. 5), we further predict that sea-level rise will induce habitat changes that will initially favor *Uca* and then *Sesarma* and *Carcinus*.

### Temporal trends

Localized recreational overfishing is causing *Sesarma*-induced creekbank vegetation loss in marshes from Long Island to Cape Cod (Coverdale, Bertness & Altieri, 2013b). However, sea-level rise is also causing creekbank vegetation loss independent of *Sesarma* grazing pressure (Schultz, Anisfeld & Hill, 2016). In RI, we have documented creekbank dieback in marshes where *Uca* is very abundant and *Sesarma* abundance has not changed over time (e.g., Coggeshall Marsh; 2014 *Uca* and *Sesarma* data from Fig. 3; 2007 *Sesarma* data from Holdredge, Bertness & Altieri, 2009), and in marshes that are distant to boat marinas and have minimal observed recreational fishing (e.g., Nag Marsh). These inconsistent findings emphasize that, as a first step, additional factors that can potentially affect crab abundance and creekbank vegetation loss need to be identified. At the mesoscale of Narragansett Bay, results from our trend analyses demonstrate that increases in crab abundance and trends of creekbank vegetation loss actually coincide with a decrease in overall recreational fishing pressure on crab-eating fish. This is also coincident with good stock status for the species identified in this study, and locally stable to increasing populations in Narragansett Bay. This negative fishing pressure finding cannot directly translate to the smaller scale of individual marshes, but within this context of an overall Bay-wide decline in fishing, it seems unlikely that fishing intensity has actually increased near marshes coincident with the onset of recent creekbank vegetation loss.

Instead, increases in crab abundance and creekbank vegetation loss in Narragansett Bay have coincided with accelerating sea-level rise and recent extreme high-water levels (Boon, 2012; Goddard et al., 2015). Sea-level rise has already been linked to an intra-marsh *Uca* range expansion (Luk & Zajac, 2013), and it can directly cause creekbank vegetation loss in the absence of crab impacts (Schultz, Anisfeld & Hill, 2016). These multiple lines of evidence agree with recent findings across southern New England (Schultz, Anisfeld & Hill, 2016; Crotty, Angelini & Bertness, 2017) and identify sea-level rise as a potential driver of creekbank vegetation loss in RI, either directly through inundation stress or indirectly via enhanced crab abundance and impacts.

**Figure 7:**
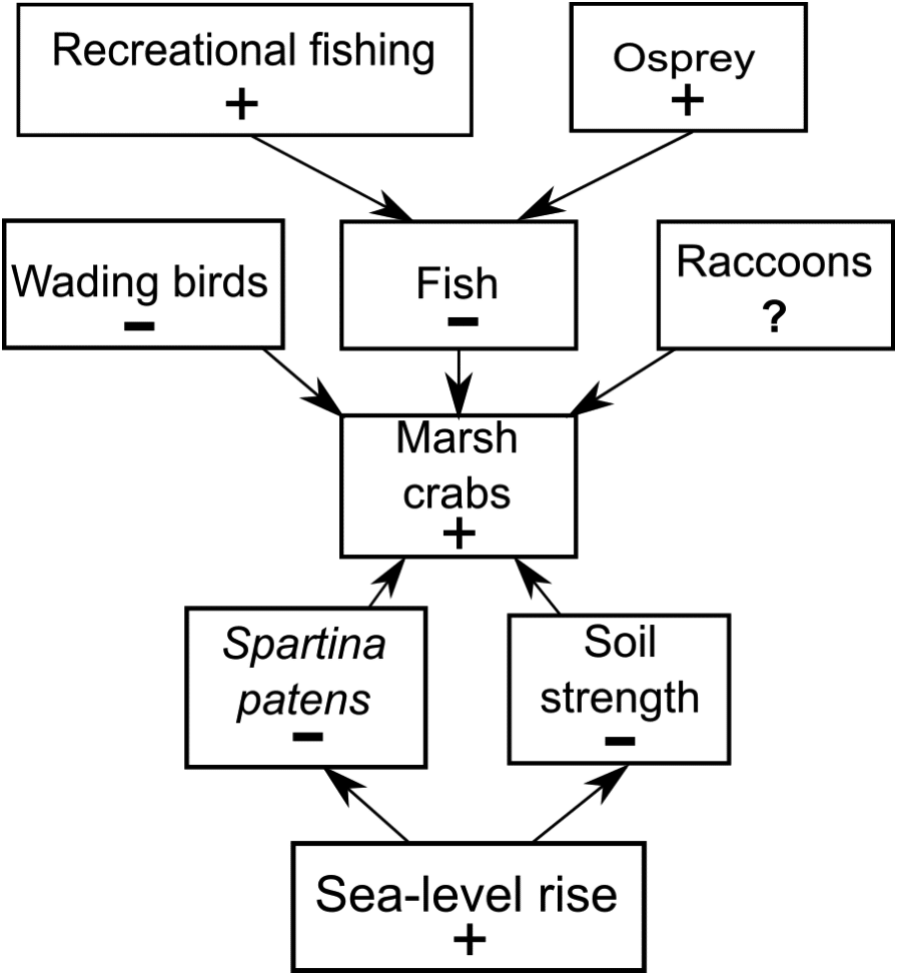
Conceptual model of marsh crab responses to the interactive effects of bottom-up (sea-level rise) and top-down (predation) processes. Positive and negative changes in each parameter are indicated by “+” and “−”, respectively (e.g., as sea-level rise increases (+), *Spartina patens* and soil strength decline (−), eliciting an increase in crabs (+). For simplicity, marsh crabs are combined into one overarching group; individual species may respond differently to changes in any single model parameter. Responses should be strongest at intermediate elevation marshes. Predation effects will be mitigated at lower elevation marshes where burrowing is inhibited by weak and waterlogged soils, and at higher elevation marshes where burrowing and activity is limited by infrequent flooding and dense high marsh vegetation.

Our trend analyses show that increases in crabs and decreases in vegetation have also coincided with changes in the abundance of direct crab consumers and predators of crab consumers. Wading birds can feed on marsh crabs (e.g., Grimes et al., 1989), and their abundance has been in steady decline in RI for almost two decades. However, we suspect that wading bird impacts on crab abundance are minimal because (1) marsh crabs are typically minor components of most wading bird diets, which are instead typically dominated by other crustaceans and/or fish (e.g., Smith, 1997; McCrimmon, Ogden & Bancroft, 2011), and (2) to our knowledge, reports of impacts to marshes from excessive crabs did not occur when wading bird abundance in RI was very low before a gradual recovery began in the 1970s (Ferren & Myers, 1998). Marsh crabs are also consumed by some estuarine fish (e.g., striped bass), which in turn serve as prey for higher-order predators, such as the Osprey. Osprey abundance has risen steadily in RI over the last four decades, largely due to expansion of human-built platforms and cell-phone towers (Walsh, 2013). Ospreys commonly nest along estuarine shores, often in or near salt marshes, and the occupants of a single osprey nest can consume over five large fish per day (McLean & Byrd, 1991). Individually, these predators may have minimal impacts on crab abundance, but their collective impacts point to the potential importance of top-down predation (potentially including fishing) as a driver of increasing crabs and creekbank vegetation loss that warrants further study.

We do not discount the effects of localized fishing at some marshes (Coverdale, Bertness & Altieri, 2013b), but our data combined with new findings (Schultz, Anisfeld & Hill, 2016) suggest a more complex scenario (Fig. 7). We hypothesize that the recent increases in crab abundance and marsh vegetation loss are the consequence of a combination of bottom-up (sea-level rise) and top-down (predation) factors and that the relative importance of each factor varies across the region and locally among individual marshes. We also acknowledge that other factors may be important beyond those we focused on here. Although data are currently lacking, examples include potential changes in predation pressure on larval crabs by planktivorous fish (Morgan, 1990), or on adult crabs by mesopredators such as raccoons (Montague, 1980; Nomann & Pennings, 1998), as well as potential changes in food availability and intra- and inter-specific competition among crabs.

## Conclusions

Our study provides new insight into the complex relationships between bottom-up and top-down stressors, crab populations, and ongoing marsh vegetation loss. Results from our four marshes demonstrate that *Uca* remains overwhelmingly dominant in RI marshes and is responding favorably to changing environmental conditions linked to increased inundation and sea-level rise. We posit that increasing crab populations are not being caused primarily by recreational overfishing; instead, sea-level rise and changing predator populations may play a more important role. At a minimum, these collective findings call for studies to further examine (1) the cause and effects of overabundant *Uca* populations on marshes, and (2) inter-marsh variability in the relative impacts of fishing, sea-level rise, and predation on crab abundance. We also recommend that ongoing long-term marsh monitoring programs be augmented to include indicators of crab abundance in order to track changes over time. The factors that drive crab overabundance and marsh vegetation loss are site-specific to some degree. It follows then that if a marsh is showing signs of degradation over time, managers will need site-specific data in order to select and implement appropriate intervention strategies. If sea-level rise is the primary driver of increasing crab abundance, managers can choose from multiple strategies aimed at building resilience that are already being evaluated (Wigand et al., 2017); if higher-order predators or localized overfishing is the driver, new management strategies will need to be identified.

## Additional Information and Declarations

### Data Availability

The following information was supplied regarding data availability: Code and data are available via GitHub (https://github.com/jhollist/crabs) and archived on Zenodo: Jeffrey W Hollister. (2018, February 23). jhollist/crabs: Code and Review Response for Edited PeerJ Manuscript (Version v2). Zenodo. http://doi.org/10.5281/zenodo.1183777.

## References

[ref-1] Altieri AH, Bertness MD, Coverdale TC, Herrmann NC, Angelini C (2012). A trophic cascade triggers collapse of a salt-marsh ecosystem with intensive recreational fishing. Ecology.

[ref-2] Atlantic States Marine Fisheries Commission (ASMFC) (2015a). Atlantic striped bass stock assessment update 2015. A report prepared by the striped bass technical committee for the Atlantic striped bass management board.

[ref-3] Atlantic States Marine Fisheries Commission (ASMFC) (2015b). Tautog benchmark stock assessment and peer review reports.

[ref-4] Belknap DF, Wilson KR (2014). Invasive European Green Crab impacts on salt marshes in Maine—sudden increase in erosion potential.

[ref-5] Bertness MD (1985). Fiddler crab regulation of *Spartina alterniflora* production on a New England salt marsh. Ecology.

[ref-6] Bertness MD, Brisson CP, Bevil MC, Crotty SM (2014). Herbivory drives the spread of salt marsh die-off. PLOS ONE.

[ref-7] Bertness MD, Coverdale TC (2013). An invasive species facilitates the recovery of salt marsh ecosystems on Cape Cod. Ecology.

[ref-8] Bertness MD, Holdredge C, Altieri AH (2009). Substrate mediates consumer control of salt marsh cordgrass on Cape Cod, New England. Ecology.

[ref-9] Bertness MD, Miller T (1984). The distribution and dynamics of *Uca pugnax* (Smith) burrows in a New England salt marsh. Journal of Experimental Marine Biology and Ecology.

[ref-10] Bigelow HB, Schroeder WC (1953). Fishes of the Gulf of Maine. U.S. Fish and Wildlife Service Bulletin.

[ref-11] Boon JD (2012). Evidence of sea level acceleration at U.S. and Canadian tide stations, Atlantic coast, North America. Journal of Coastal Research.

[ref-12] Brisson CP, Coverdale TC, Bertness MD (2014). Salt marsh die-off and recovery reveal disparity between the recovery of ecosystem structure and service provision. Biological Conservation.

[ref-13] Chang W (2009). R graphics cookbook: practical recipes for visualizing data.

[ref-14] Cole Ekberg M, Ferguson W, Raposa K (2015). Results of the 1st Rhode Island salt marsh assessment: final report. Final report to the Rhode Island Coastal and Estuarine Habitat Restoration Trust Fund.

[ref-15] Coverdale TC, Bertness MD, Altieri AH (2013b). Regional ontogeny of New England salt marsh die-off. Conservation Biology.

[ref-16] Coverdale TC, Brisson CP, Young EW, Yin SF, Donnelly JP, Bertness MD (2014). Indirect human impacts reverse centuries of carbon sequestration and salt marsh accretion. PLOS ONE.

[ref-17] Coverdale TC, Herrmann NC, Altieri AH, Bertness MD (2013a). Latent impacts: the role of historical human activity in coastal habitat loss. Frontiers in Ecology and the Environment.

[ref-18] Crotty SM, Angelini C, Bertness MD (2017). Multiple stressors and the potential for synergistic loss of New England salt marshes. PLOS ONE.

[ref-19] Deegan LA, Johnson DS, Warren RS, Peterson BJ, Fleeger JW, Fagherazzi S, Wollheim WM (2012). Coastal eutrophication as a driver of salt marsh loss. Nature.

[ref-20] Elmer WH, Useman S, Schneider RW, Marra RE, LaMondia JA, Mendelssohn IA, Jiménez-Gasco MM, Caruso FL (2013). Sudden vegetation dieback in Atlantic and Gulf coast salt marshes. Plant Disease.

[ref-21] Fanini L, Lowry JK (2016). Comparing methods used in estimating biodiversity on sandy beaches: Pitfall vs. quadrat sampling. Ecological Indicators.

[ref-22] Ferren RL, Myers JE (1998). Rhode Island’s maritime nesting birds.

[ref-23] Goddard PB, Yin J, Griffies SM, Zhang S (2015). An extreme event of sea-level rise along the Northeast coast of North America in 2009-2010. Nature Communications.

[ref-24] Grimes BH, Huish MT, Kerby JH, Moran D (1989). Species profiles: life histories and environmental requirements of coastal fishes and invertebrates (mid-Atlantic): Atlantic marsh fiddler. U.S. Fish and Wildlife Service biological report 82 (11.114).

[ref-25] Heiri O, Lotter AF, Lemcke G (2001). Loss on ignition as a method for estimating organic and carbonate content in sediments: reproducibility and comparability of results. Journal of Paleolimnology.

[ref-26] Holdredge C, Bertness MD, Altieri AH (2009). Role of crab herbivory in die-off of New England salt marshes. Conservation Biology.

[ref-27] Hollister JW, Raposa KB (2017). jhollist/crabs: code for manuscript submission. Zenodo.

[ref-28] Hughes ZJ, FitzGerald DM, Wilson CA, Pennings SC, Więski K, Mahadevan A (2009). Rapid headward erosion of marsh creeks in response to relative sea level rise. Geophysical Research Letters.

[ref-29] Jaramillo E, Lunecke K (1988). The role of sediments in the distribution of *Uca pugilator* (Bosc) and *Uca pugnax* (Smith) (Crustacea, Brachyura) in a salt marsh of Cape Cod. Meeresforschung.

[ref-30] Luk YC, Zajac RN (2013). Spatial ecology of fiddler crabs, *Uca pugnax*, in southern New England salt marsh landscapes: Potential habitat expansion in relation to salt marsh change. Northeastern Naturalist.

[ref-31] McCrimmon DA, Ogden JC, Bancroft GT, Rodewald PG (2011). Great Egret (*Ardea alba*), the birds of North America.

[ref-32] McKinney RA, Wigand C (2006). A framework for the assessment of the wildlife habitat value of New England salt marshes.

[ref-33] McLean PK, Byrd MA (1991). The diet of Chesapeake Bay ospreys and their impact on the local fishery. Journal of Raptor Research.

[ref-34] Meng L, Cicchetti G, Chintala M (2004). Nekton habitat quality at shallow water sites in two Rhode Island coastal systems. Estuaries.

[ref-35] Montague CL (1980). A natural history of temperate western Atlantic fiddler crabs (genus *Uca*) with reference to their impact on the salt marsh. Contributions in Marine Science.

[ref-36] Morgan SG (1990). Impact of planktivorous fishes on dispersal, hatching, and morphology of estuarine crab larvae. Ecology.

[ref-37] NOAA (2015). Estimation of vertical uncertainties in VDatum.

[ref-38] Nomann BE, Pennings SC (1998). Fiddler crab-vegetation interactions in hypersaline habitats. Journal of Experimental Marine Biology and Ecology.

[ref-39] Northeast Fisheries Science Center (NEFSC) (2015). 60th Northeast regional stock assessment workshop (60th saw) assessment report. US Department of Commerce, Northeast Fisheries Science Center reference document. 15-08.

[ref-40] R Core Team (2017). R: a language and environment for statistical computing. https://www.R-project.org/.

[ref-41] Raposa KB, Ekberg MLC, Burdick DM, Ernst NT, Adamowicz SC (2017b). Elevation change and the vulnerability of Rhode Island (USA) salt marshes to sea-level rise. Regional Environmental Change.

[ref-42] Raposa KB, Wasson K, Smith E, Crooks JA, Delgado P, Fernald SH, Ferner MC, Helms A, Hice LA, Mora JW, Puckett B, Sanger D, Shull S, Spurrier L, Stevens R, Lerberg S (2016). Assessing tidal marsh resilience to sea-level rise at broad geographic scales with multi-metric indices. Biological Conservation.

[ref-43] Raposa KB, Weber RLJ (2013). Implementing the NERR Sentinel Sites Program at the Narragansett Bay Research Reserve to track salt marsh responses to climate change stressors. Narragansett Bay Research Reserve Technical Reports Series 2013:1.

[ref-44] Raposa KB, Weber RLJ, Ekberg MC, Ferguson W (2017a). Vegetation dynamics in Rhode Island salt marshes during a period of accelerating sea level rise and extreme sea level events. Estuaries and Coasts.

[ref-45] Revelle W (2017). https://cran.r-project.org/web/packages/psych/index.html.

[ref-46] Robinson D (2017). https://CRAN.R-project.org/package=broom.

[ref-47] Roman CT, James-Pirri MJ, Heltshe JF (2001). Monitoring salt marsh vegetation: a protocol for the long-term Coastal Ecosystem Monitoring Program at Cape Cod National Seashore.

[ref-48] Roman CT, Raposa KB, Adamowicz SC, James-Pirri MJ, Catena JG (2002). Quantifying vegetation and nekton response to tidal restoration of a New England salt marsh. Restoration Ecology.

[ref-49] Rudis B (2017). https://cran.r-project.org/web/packages/hrbrthemes/index.html.

[ref-50] Schultz RA, Anisfeld SC, Hill TD (2016). Submergence and herbivory as divergent causes of marsh loss in Long Island Sound. Estuaries and Coasts.

[ref-51] Shepherd GR, Nieland J (2010). Black sea bass 2010 stock assessment update. US Department of Commerce, Northeast fisheries science center reference document 10-13.

[ref-52] Smith JP (1997). Nesting season food habits of four species of herons and egrets at Lake Okeechobee, Florida. Colonial Waterbirds.

[ref-53] Smith SM (2015). Does loss of salt marsh vegetation caused by a native grapsid crab improve habitat suitability for the Atlantic mud fiddler (*Uca pugnax*)?. Journal of Crustacean Biology.

[ref-54] Topping CJ, Sunderland KD (1992). Limitations to the use of pitfall traps in ecological studies exemplified by a study of spiders in a field of winter wheat. Journal of Applied Ecology.

[ref-55] Vu HD, Wieski K, Pennings SC (2017). Ecosystem engineers drive creek formation in salt marshes. Ecology.

[ref-56] Walsh ES (2013). A review of thirty-five years of Osprey (*Pandion haliaetus*) nesting data in Rhode Island. Maser of Environmental Science and Management thesis.

[ref-57] Warren RS, Niering WA (1993). Vegetation change on a northeast tidal marsh: interaction of sea level rise and marsh accretion. Ecology.

[ref-58] Watson EB, Raposa KB, Carey JC, Wigand C, Warren RS (2017b). Anthropocene survival of southern New England’s salt marshes. Estuaries and Coasts.

[ref-59] Watson EB, Szura K, Wigand C, Raposa KB, Blount K, Cencer M (2016). Sea level rise, drought and the decline of *Spartina patens* in New England marshes. Biological Conservation.

[ref-60] Watson EB, Wigand C, Davey EW, Andrews HM, Bishop J, Raposa KB (2017a). Wetland loss patterns and inundation-productivity relationships prognosticate widespread salt marsh loss for southern New England. Estuaries and Coasts.

[ref-61] Wickham H (2016). ggplot2: elegant graphics for data analysis.

[ref-62] Wickham H (2017). https://CRAN.R-project.org/package=forcats.

[ref-63] Wickham H, Bryan J (2017). https://CRAN.R-project.org/package=readxl.

[ref-64] Wickham H, Francois R, Henry L, Müller K (2017a). https://CRAN.R-project.org/package=dplyr.

[ref-65] Wickham H, Henry L (2017). https://CRAN.R-project.org/package=tidyr.

[ref-66] Wickham H, Hester J, Francois R (2017b). https://CRAN.R-project.org/package=readr.

[ref-67] Wigand C, Ardito T, Chaffee C, Ferguson W, Paton S, Raposa K, Vandemoer C, Watson E (2017). A climate change adaptation strategy for management of coastal marsh systems. Estuaries and Coasts.

[ref-68] Wigand C, McKinney RA, Charpentier MA, Chintala MM, Thursby GB (2003). Relationships of nitrogen loadings, residential development, and physical characteristics with plant structure in New England salt marshes. Estuaries.

[ref-69] Wigand C, McKinney R, Chintala M, Lussier S, Heltshe J (2010). Development of a reference coastal wetland set in Southern New England (USA). Environmental Monitoring and Assessment.

[ref-70] Wigand C, Roman CT, Davey E, Stolt M, Johnson R, Hanson A, Watson EB, Moran SB, Cahoon DR, Lynch JC, Rafferty P (2014). Below the disappearing marshes of an urban estuary: historic nitrogen trends and soil structure. Ecological Applications.

[ref-71] Wilson CA, Hughes ZJ, FitzGerald DM (2012). The effects of crab bioturbation on Mid-Atlantic saltmarsh tidal creek extension: geotechnical and geochemical changes. Estuarine, Coastal and Shelf Science.

[ref-72] Yang Z, Hess K, Wong A, Spargo E, White S, Myers E (2008). VDatum for the Long Island Sound, Narragansett Bay, and New York Bight: tidal datums, marine grids, and sea surface topography. NOAA Technical Report NOS.

